# Hemodynamic performance within crossed stent grafts: computational and experimental study on the effect of cross position and angle

**DOI:** 10.1186/s12938-018-0517-1

**Published:** 2018-06-19

**Authors:** Ming Liu, Anqiang Sun, Xiaoyan Deng

**Affiliations:** 1 Key Laboratory for Biomechanics and Mechanobiology of Ministry of Education, School of Biological Science & Medical Engineering, Beihang University, Beijing, 100083 China; 2 Beijing Advanced Innovation Centre for Biomedical Engineering, Beihang University, Beijing, 102402 China

**Keywords:** Bifurcated stent graft, Hemodynamic, Helical flow, Migration

## Abstract

**Background and aims:**

The crossed limbs stent graft technique is regularly employed to treat abdominal aortic aneurysm patients with unfavorable aneurysm necks or widely splayed common iliac arteries. This article numerically evaluates the hemodynamic performance of the crossed limbs strategy by analyzing numerical simulations and conducting experiments using two series of idealized bifurcated stent grafts with different cross angles and cross positions.

**Results:**

Results demonstrated that the absolute helicity at outlets decreased with increased cross angles and increased with decreased cross positions. The time-averaged wall shear stress remained approximately unchanged, whereas the oscillating shear index and relative resident time decreased slightly when the cross angle increased and cross position decreased in iliac grafts. Additionally, both numerical and in vitro experimental results indicate the displacement force acting on the stent graft gradually increased as cross angles increased and cross positions decreased. Results further indicated that strip areas with a high oscillating shear index and high relative resident time, which may be vulnerable to thrombosis formation, exist along the outer surface of the iliac artery grafts.

**Conclusion:**

Given this information, the optimal crossed limbs configuration may contain a small cross angle and low cross position; however, low cross positions may increase the risk of migration.

## Background

Clinically, bifurcated stent grafts comprised of modular components that allow graft-guided blood flow to the two iliac arteries are most commonly used in endovascular aneurysm repair (EVAR) [1, 2]. The conventional surgery procedure employs proximal neck deployment (with an attached ipsilateral iliac limb) first, followed by contralateral iliac limb deployment. This conventional EVAR procedure becomes more complex and difficult when abdominal aortic aneurysm (AAA) patients have an unfavorable anatomy such as widely splayed common iliac arteries or severe aneurysm necks [3, 4]. Surgeons sometimes employ the “crossed limbs” technique to overcome such problems, in which the limbs of a bifurcated stent graft (BSG) are rotated into the so-called “ballerina position” at the discretion of the surgeon [4–6].

Several studies have reported that the migration force acting on the stent graft due to blood flow is much lower for the crossed limbed strategy relative to the conventional strategy [5, 7]. The crossed limbs strategy generates helical flows within the iliac artery grafts [5, 6], resembling patterns typically observed in the human aorta [8, 9]. Helical flow patterns have been documented to suppress atherogenic lipid deposition within the arterial wall, enhance oxygen supply to arteries, and reduce platelet adhesion [10, 11], thereby protecting the arterial wall from atherosclerosis and thrombosis formation [8, 12–15]. The crossed limbs strategy is thus believed to be advantageous for AAA repair treatment.

**Fig. 1:**
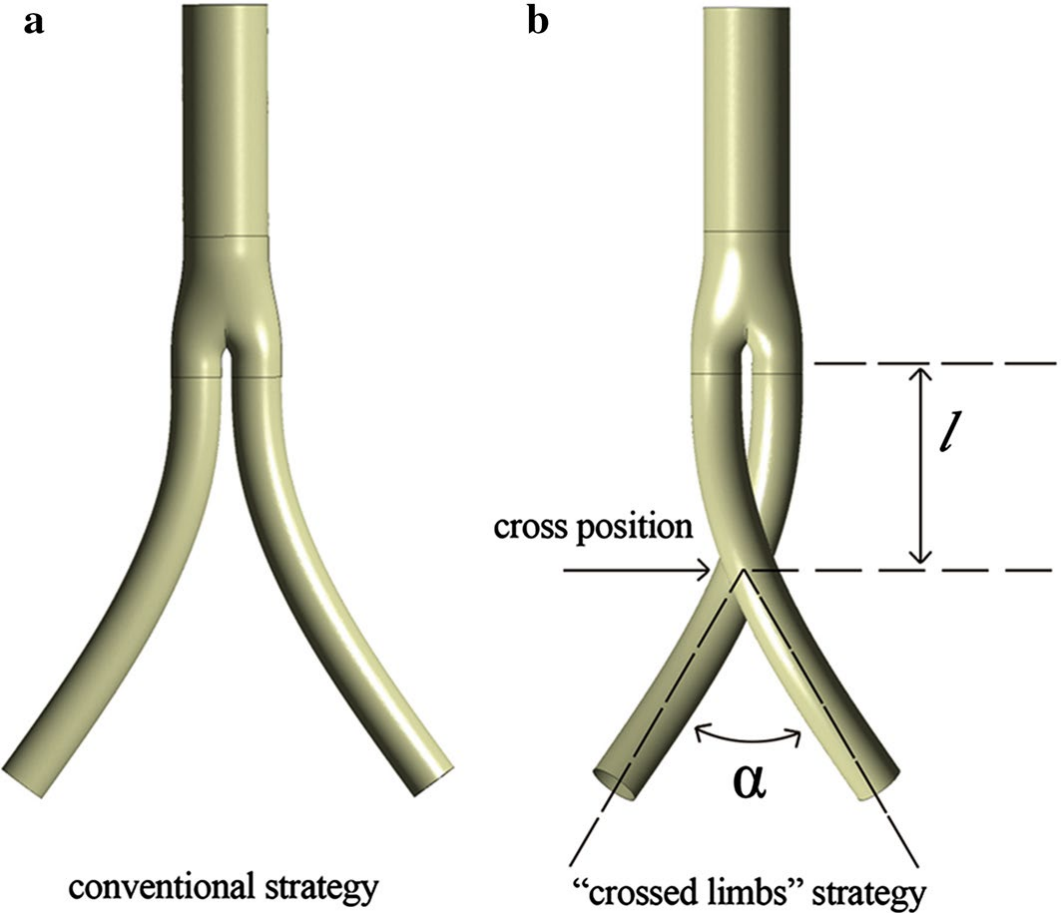
Two typical bifurcated stent graft strategies used in EVAR **a** conventional; **b** “crossed limbs” (cross position *l* refers to the length from the bifurcation point to the cross point, and cross angle *α* refers to the angle between the two bilateral limb grafts)

As depicted in Fig. 1, two geometrical parameters may influence the hemodynamic performance of the crossed limbs strategy: the cross angle *α* and cross position *l*. Although a few preliminary studies comparing the crossed limbs and conventional strategies have revealed the hemodynamic benefits of crossed limbs [4–7, 16–19], no investigation has been conducted regarding the optimal deployment arrangement for the crossed limbed strategy in terms of the cross angle and position. Therefore, this study performed computational fluid dynamic (CFD) simulations and in vitro experiments on this strategy using idealized models to determine the optimal parameters for both the cross angle *α* and cross position *l*.

## Methods

### Geometry and meshing

**Fig. 2:**
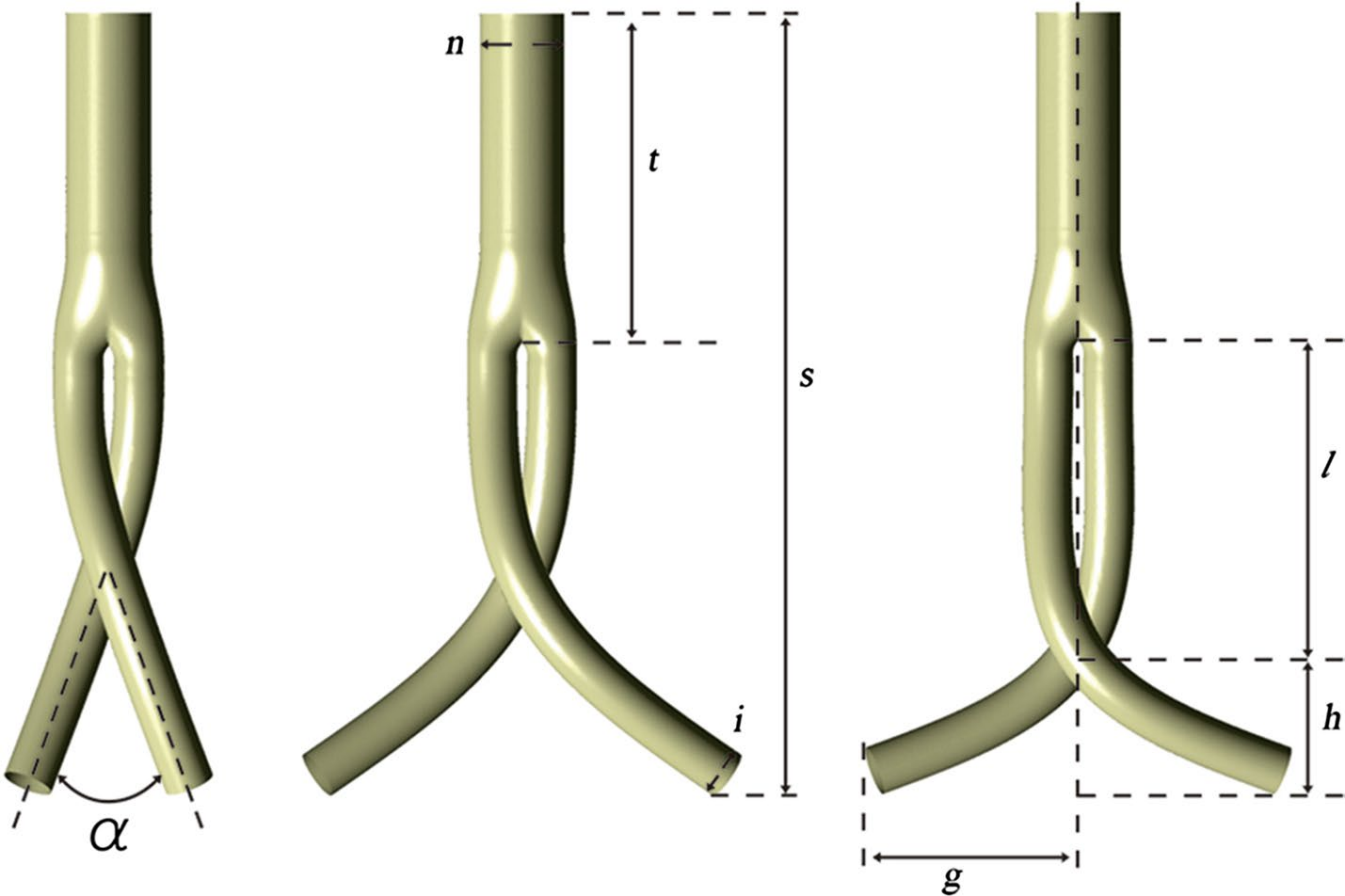
Ideal bifurcated stent grafts in terms of various cross angles and positions. *s* refers to the stent graft length, *t* the trunk stent graft length, *n* the neck diameter, *i* the iliac diameter, *α* the cross angle of iliac grafts, *l* the distance from bifurcation point to the cross point, *h* the distance from the cross point to the bottom plane, and *g* the side length of iliac graft

Two series of idealized BSGs with iliac artery grafts positioned at various cross angles and positions were generated for this study, as shown in Fig. 2, based on the parameters presented in literature [18, 19] using the commercial software SolidWorks (Solid Works Corp, Concord, MA). All configurations shared an identical stent graft trunk body. The total axis direction length of the grafts was 154.64 mm, the trunk comprising 71 mm. The trunk and iliac graft diameters were 17 and 10 mm, respectively. The cross angle of the BSG was defined as the angle (α) between the iliac limbs and set to 30°, 45°, 60°, 75°, and 90°, among the models. The length *l* remained constant at 42 mm. The relative cross position was defined as the ratio of length *l* to length *h* and set to 0.48, 0.72, 1, 1.4, and 1.92. The side length *g* remained 41 mm regardless of the cross position.

Computational meshes were modeled using ANSYS ICEM CFD (ANSYS Inc., Canonsburg, PA). Surface meshes contained a mixture of tetrahedral and hexahedral volume meshes. The maximum and minimum mesh sizes were 0.8 and 0.05 mm, respectively. Each mesh contained approximately 1.7 million cells.

### Governing equations

Flow simulations were based on the respective conservation equations for mass and momentum, which are defined:

1 $$ \rho \left( {\left( {{{\partial {\mathbf{u}}} \mathord{\left/ {\vphantom {{\partial {\mathbf{u}}} {\partial t}}} \right. \kern-0pt} {\partial t}}} \right) + \left( {{\mathbf{u}} \cdot \nabla } \right){\mathbf{u}}} \right) = - \nabla {\text{p}} + \nabla \tau $$

2 $$ \nabla \cdot {\mathbf{u}} = 0 $$

where **u** and *p* represent, respectively, the fluid velocity vector and pressure; ρ is the blood density (ρ = 1050 kg/m^3^); and τ denotes the stress tensor:

3 $$ \uptau = 2\upeta\left( {{\dot{\gamma }}} \right){\mathbf{D}} $$

Where **D** is the strain rate tensor defined as ${\mathbf{D}}({\mathbf{u}}){ \,=\, }\frac{1}{2}\left( {\nabla {\mathbf{u}}{ + }\nabla {\mathbf{u}}^{T} } \right)$ and $\dot{\gamma } = \sqrt {\text{2}{\mathbf{D}}:{\mathbf{D}}}$ is the strain rate tensor modulus.

The non-Newtonian nature of the flow was accounted for by using the Carreau model, written as [20]

4 $$ \eta \left( {\dot{\gamma }} \right) = \eta_{\infty } + (\eta_{0} - \eta_{\infty } )\left[ {1 + \left( {\lambda \dot{\gamma }} \right)^{2} } \right]^{{\left( {\left( {n - 1} \right)/2} \right)}} $$

where *η*_0_ and *η*_∞_ are the zero and infinite shear rate viscosities, respectively, and *λ* is the relaxation time constant. The Carreau model was found to fit the experimental data well [21], where *η*_∞_ = 3.45 × 10^−3^ Pa s, *η*_0_ = 5.6 × 10^−2^ Pa s, n = 0.3568, and *λ *= 3.313 s.

### Boundary conditions

**Fig. 3:**
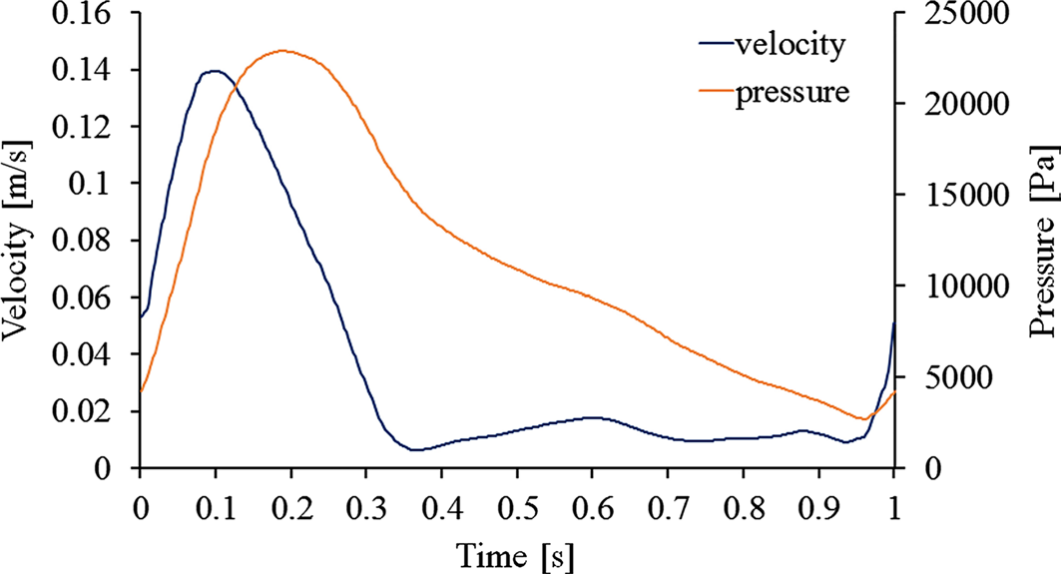
Imposed inlet velocity and outlet pressure waveforms

First, steady flow simulations were performed for all cases. This solution was used as the initial iteration data for further pulsatile flow simulations. For the steady flow simulations, a mean velocity of 0.044 m/s was set at the inlet and constant pressure of 13,300 Pa was set at outlets. For the pulsatile flow simulation, the time-dependent flat flow velocity waveform shown in Fig. 3 was set at the inlet, and the time-dependent pressure waveform in Fig. 3 was assigned at the outlets [16]. The BSG wall was assumed to be rigid and non-slippery.

### Numerical scheme

The finite volume method was adopted to solve the mass and momentum conservation equations using ANSYS Fluent CFD (ANSYS Inc., Canonsburg, PA). These calculations were performed in 200-step cycles, with a step time of 0.005 s. A pressure-based solver was used with a second-order upwind scheme for momentum spatial discretization. The residual continuity and velocity were both assigned values of 1.0 × 10^−5^. Five pulsatile cycles were computed to obtain a periodic solution, and a sixth was used as the final solution. MATLAB (MathWorks) and Tecplot (Tecplot) were used in post-processing to analyze data and observe results.

### Quantities of interest

Helicity measures the alignment/misalignment of the local velocity and vorticity vectors, and the sign of helicity indicates the rotating direction of helical structures [22]. *H*_*d*_, the helicity density, is defined by Eq. (5) [23, 24]:

5 $$ \text{H}_{\text{d}} \text{ = }{\mathbf{u}} \cdot \left( {\nabla \times {\mathbf{u}}} \right) . $$

Hemodynamic indicators based on wall shear stress, including the time-averaged wall shear stress (TAWSS), oscillating shear index (OSI) and relative resident time (RRT), were calculated for unsteady simulations. The shear stress on the stent graft throughout a cardiac cycle was evaluated using the TAWSS based on the following equation:

6 $$ \text{TAWSS = }\frac{\text{1}}{T}\int_{0}^{\text{T}} {\left| {{\mathbf{WSS}}} \right|} \;{\text{dt}} $$

where T is the lasting time of a pulsatile cycle, wall shear stress (WSS) is the instantaneous wall shear stress vector, and s is the position on the stent graft wall.

The OSI, described in Eq. 7 [25], was used to reflect directional variations in the WSS vector during a pulsatile cycle, with higher values occurring particularly in regions characterized by disturbed flow.

7 $$ \text{OSI}=\frac {1}{2}\left[ {1 - \left( {\frac{{\left| {\int_{{0}}^{T} {{\mathbf{WSS}}}\;\text{dt}} \right|}}{{\int_{{0}}^{T} {\left| {{\mathbf{WSS}}} \right|\text{dt}} }}} \right)} \right] $$

The RRT was used to evaluate the resident time of the blood flow and indicate regions suffering both low and high oscillating WSS, with the RRT index quantified as follows:

8 $$ \text{RRT = }\frac{\text{1}}{{\left( {1 - 2 \cdot \text{OSI}} \right) \cdot \text{TAWSS}}} $$

## Results

### Flow pattern

**Fig. 4:**
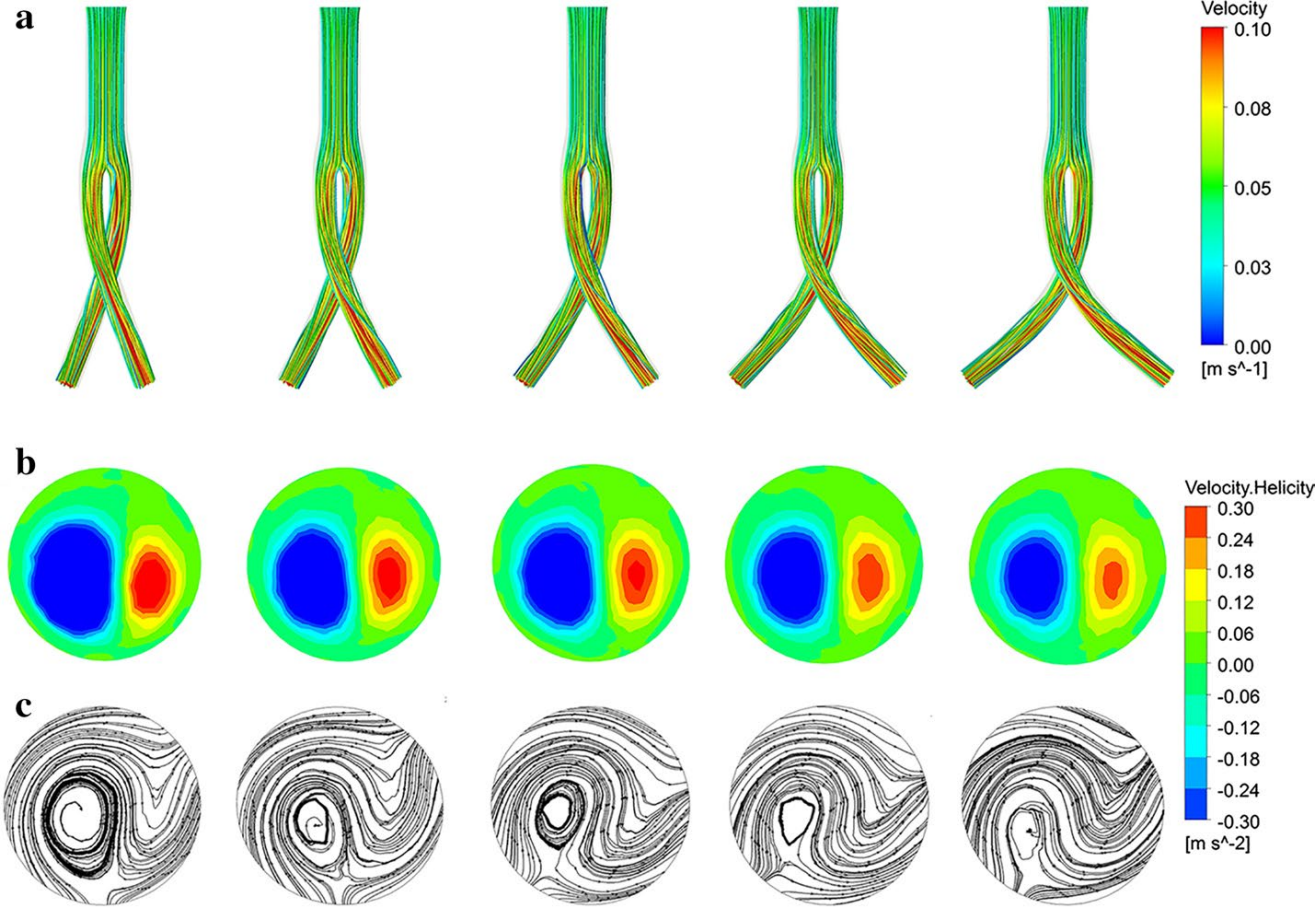
Influence of cross angle on flow pattern within the stent graft under steady-state. From left to right, cross angles are 30°, 45°, 60°, 75°, and 90° respectively. **a** Streamlines colored by the magnitude of velocity; **b** contours of helicity at the left iliac graft outlet; **c** surface streamlines at the left iliac graft outlet

**Fig. 5:**
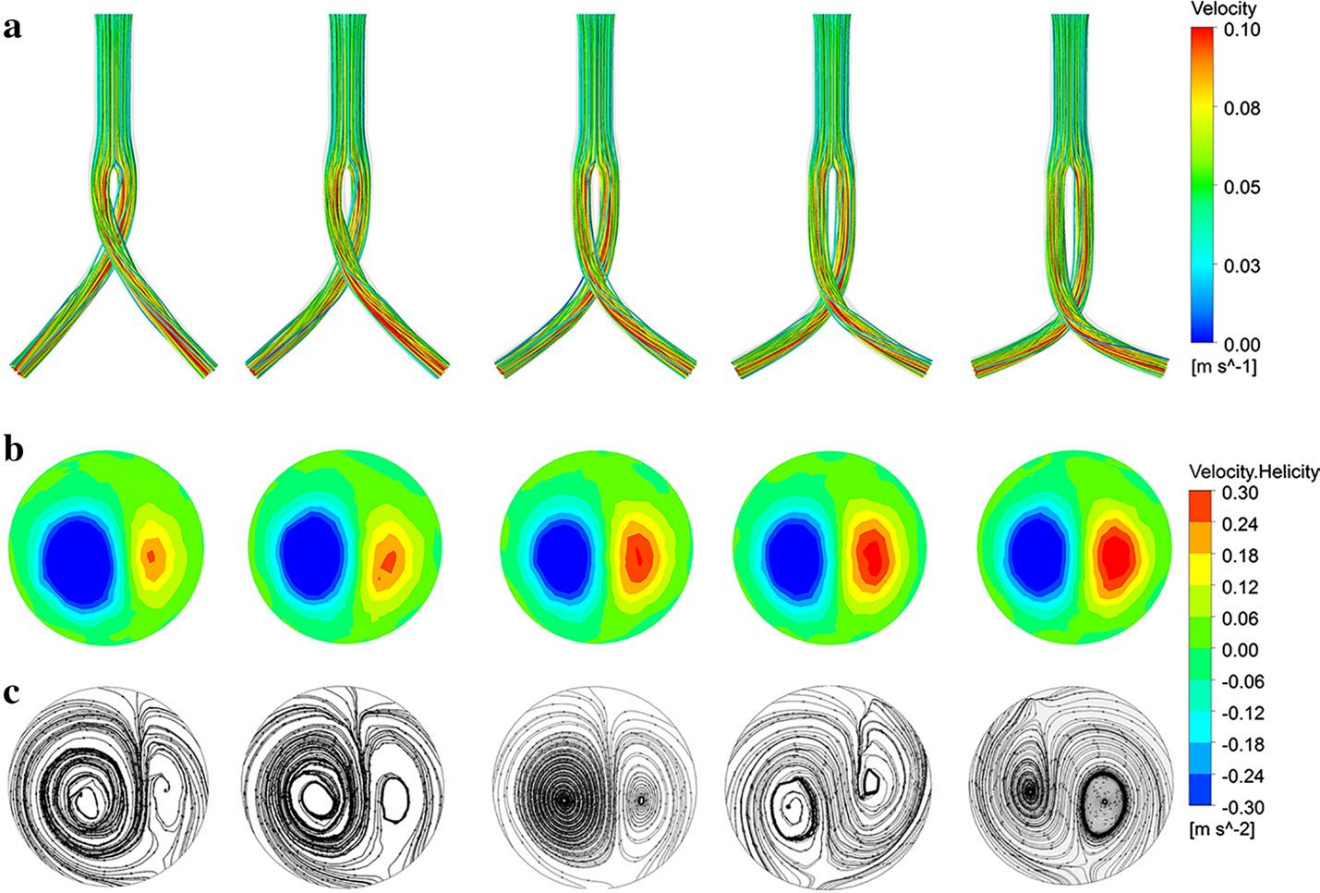
Influence of cross position on flow pattern within the stent graft under steady-state. From left to right, cross position ratios are 0.48, 0.72, 1, 1.4, and 1.92 respectively. **a** Streamlines colored by the magnitude of velocity; **b** contours of helicity at the left iliac graft outlet; **c** surface streamlines at the left iliac graft outlet

The flow pattern can be visualized as three-dimensional streamlines. Streamlines within the iliac grafts have higher velocities relative to the trunk graft (Figs. 4a, 5a). The streamlines gradually twisted along the iliac grafts, with double helical flows observed at the left iliac graft outlet. As the idealized models in this study were symmetrical, the two iliac graft outlets presented similar phenomena. When considering the cross angle effect (Fig. 4), the observed left-handed helical flow was much larger than the right-handed one (Fig. 4b, c). As the crossed angle gradually increased, the sizes of these two helical flows became similar. Considering the cross position effect, observed left-handed helical flows were also much larger than right-handed ones (Fig. 5b, c). As the position ratio increased, associated with lower cross positions, the left-handed helical flow progressively decreased whereas the right-handed helical flow increased.

**Fig. 6:**
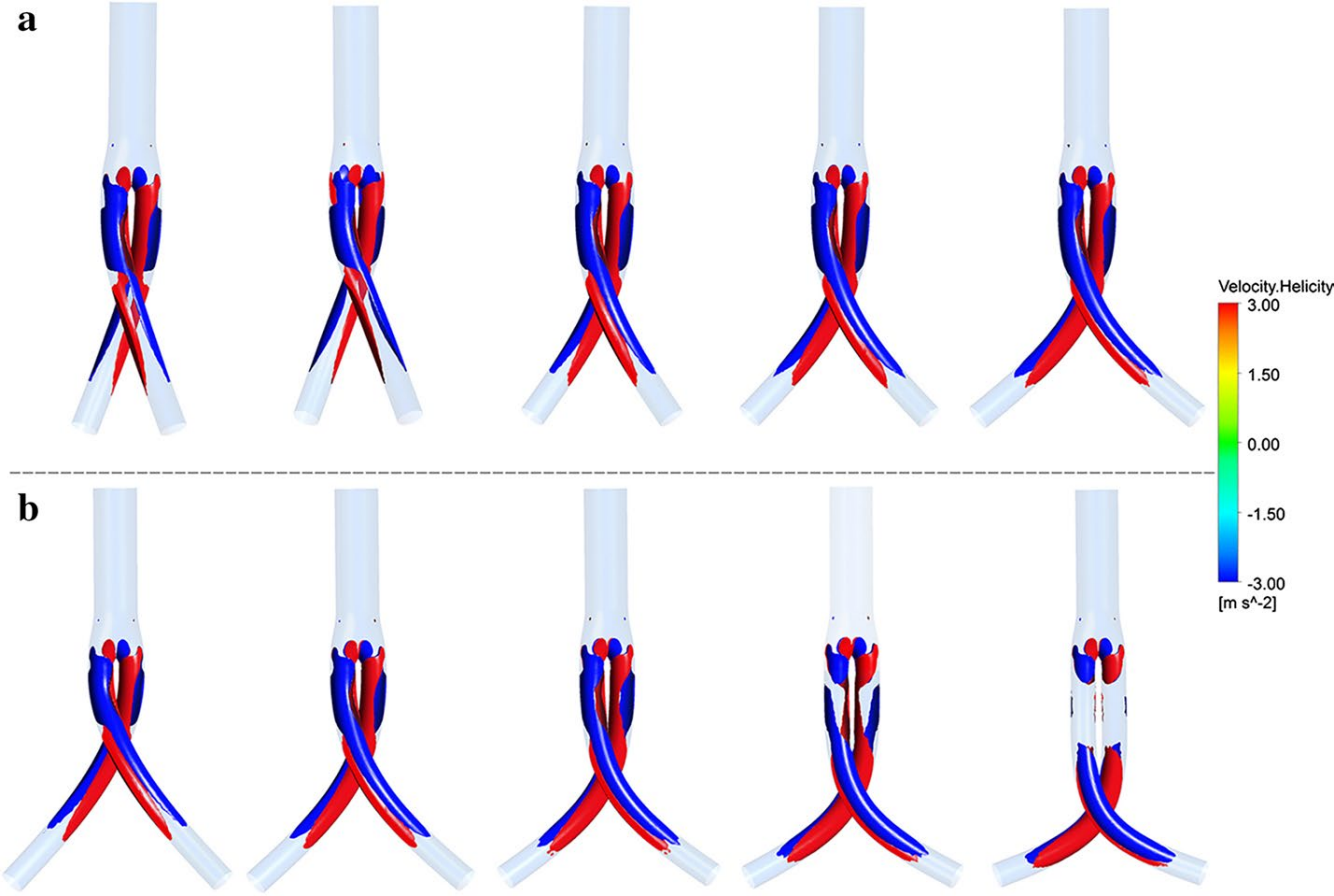
Helicity iso-surfaces at peak systole (t = 0.1 s) from pulsatile flow computations. From left to right, **a** cross angles of 30°, 45°, 60°, 75°, 90°. **b** Cross position ratios of 0.48, 0.72, 1, 1.4, 1.92

Positive (red color) and negative (blue color) helicity values indicate right-handed and left-handed rotating fluid structures along the iliac artery grafts, respectively. Figure 6 depicts the − 3/3 m s^−2^ helicity iso-surfaces. The two helical flow structures are shown to exist widely along the iliac grafts from the bifurcation point. Considering the effect of cross angles, the largest helical structure twist degree was observed with a cross angle of 30° (Fig. 6a). Models with cross position ratios of 1.4 and 1.92 showed helicity structures distributed within the crossed portions isolated from the bifurcation point (Fig. 6b).

### Helicity analysis

**Fig. 7:**
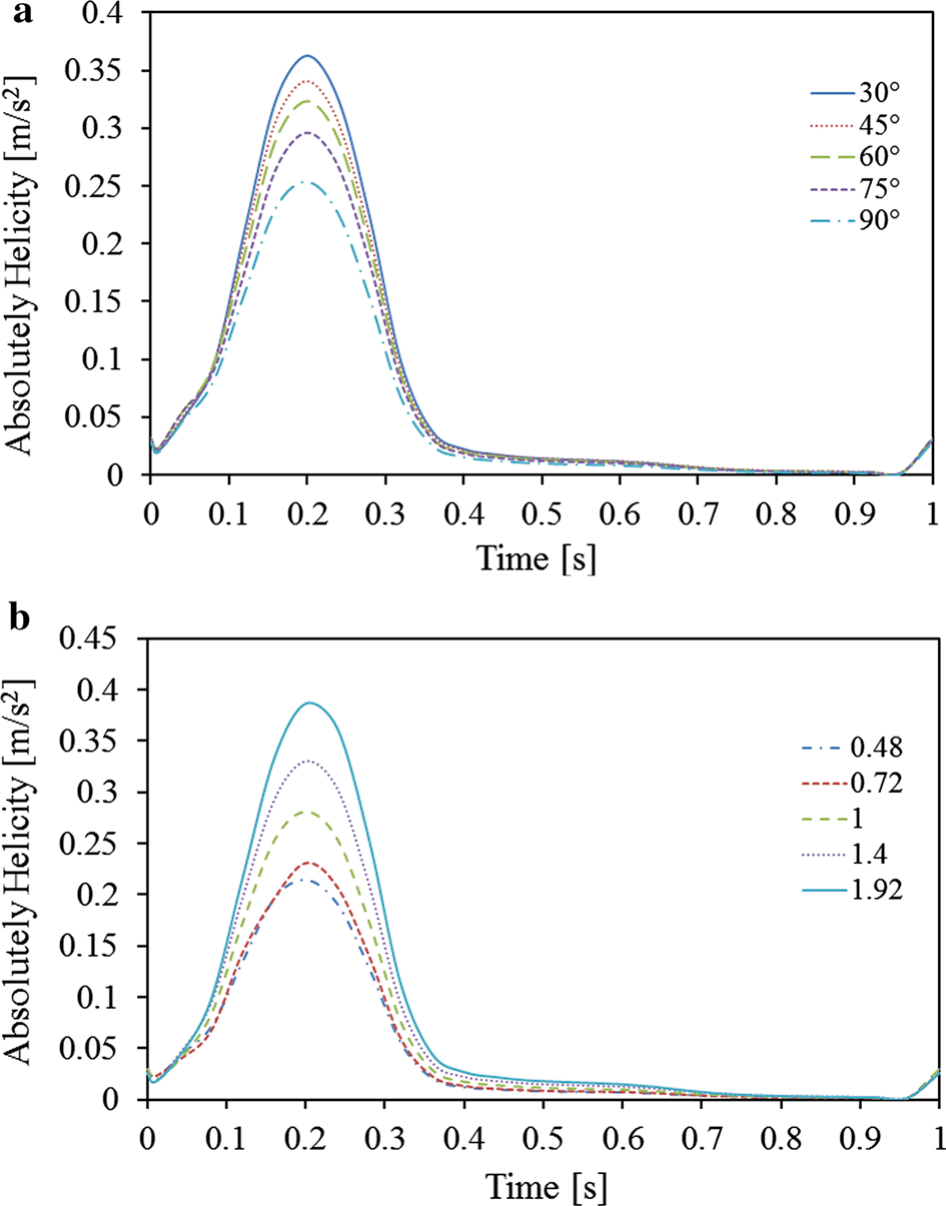
Absolute area-averaged helicity in the left iliac graft outlet. **a** Effect of cross angle; **b** effect of cross position ratio

The absolute area-averaged helicity trends of the left iliac outlet are plotted and compared in Fig. 7. The absolute helicity was obviously higher during systole than the rest of the cycle, and approached zero during diastole. As the cross angle increased, the magnitude of the absolute area-averaged helicity gradually decreased from 0.36 to 0.25 m s^−2^. The absolute area-averaged helicity increased from 0.21 to 0.39 m s^−2^ as the cross-position ratio increased.

### TAWSS, OSI, and RRT distributions

**Fig. 8:**
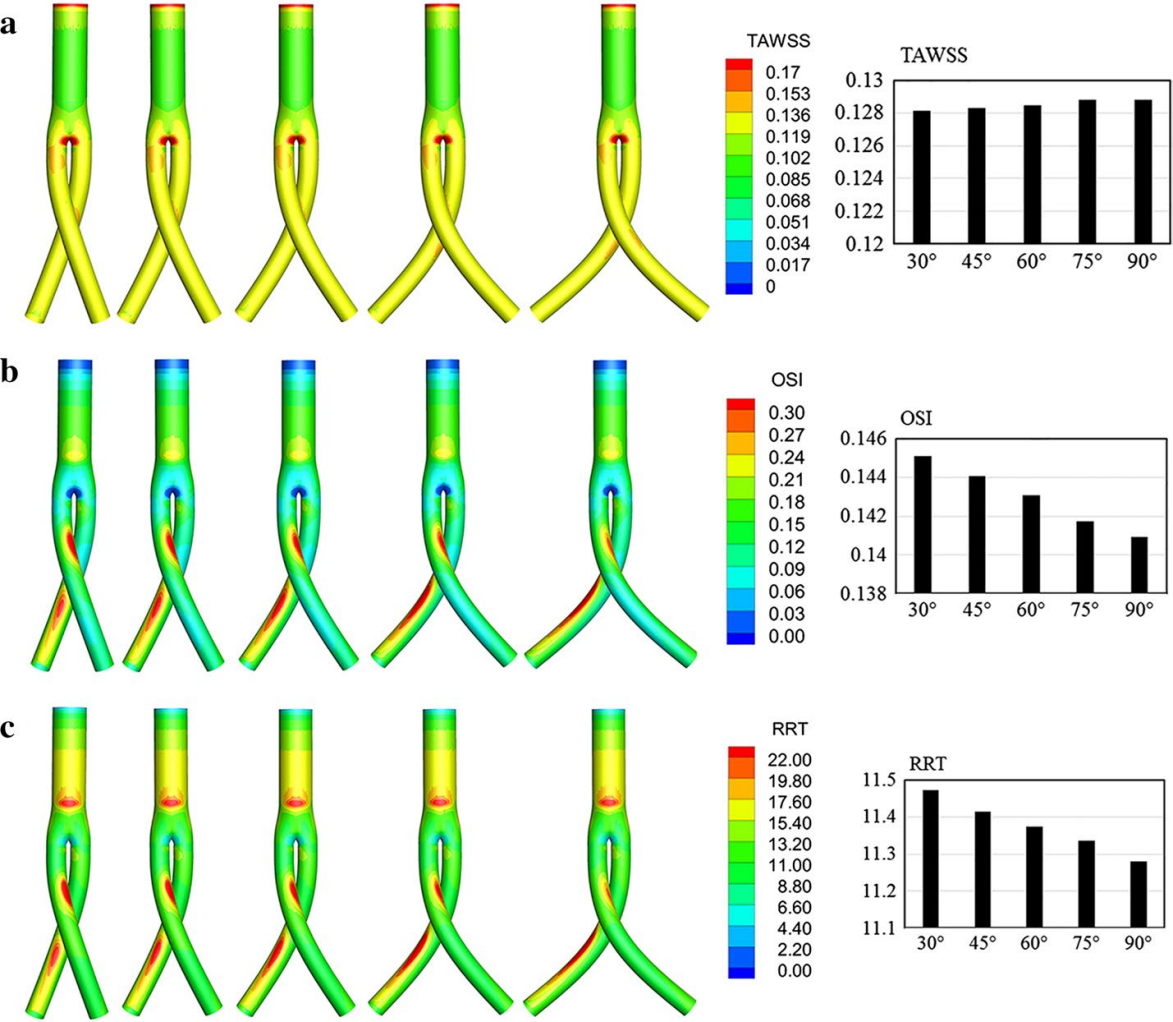
Crossed stent grafts with cross angles of 30°, 45°, 60°, 75°, and 90°, from left to right. **a** Contours of TAWSS. **b** Contours of OSI. **c** Contours of RRT. Bar graphs represent area-averaged mean of these parameters on the bilateral iliac grafts

As shown in Fig. 8a, relatively high TAWSS areas were observed in the stent graft bifurcation and cross regions. High OSI (Fig. 8b) and RRT (Fig. 8c) strip areas were observed on the outer surface of stent grafts. These hemodynamic indicators on the iliac grafts were extracted and compared in histogram form. As the cross angle increased, the area-averaged weighted TAWSS in iliac grafts remained approximately 0.128 Pa, whereas the OSI decreased from 0.145 to 0.141 and RRT decreased slightly, from 11.47 to 11.28.

**Fig. 9:**
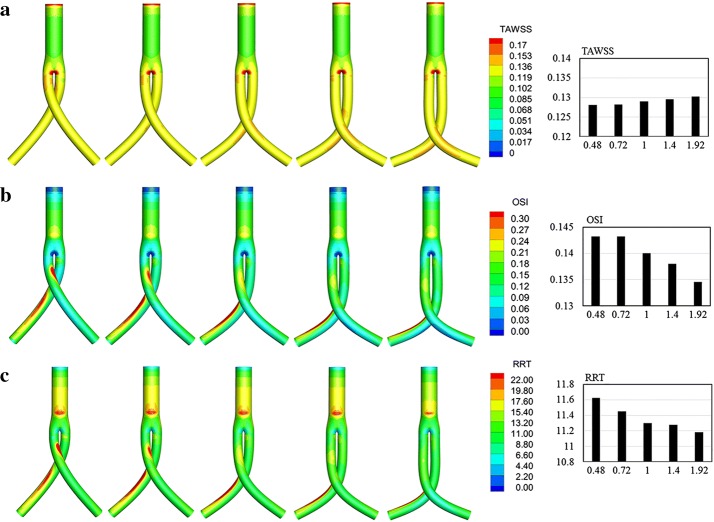
Crossed stent grafts with cross position ratios of 0.48, 0.72, 1, 1.4, and 1.92, from left to right. **a** Contours of TAWSS. **b** Contours of OSI. **c** Contours of RRT. Bar graphs represent area-averaged mean of these parameters on the bilateral iliac grafts

As shown in Fig. 9a, relatively high TAWSS areas were observed in stent graft bifurcation and cross regions. High OSI strip areas were observed on the outer surface of stent grafts, whereas low OSIs were observed on its inner surface (Fig. 9b). In Fig. 9c, high RRT strip areas can be observed on the outer surface of stent graft. When examined in combination, high OSI and RRT values appear to have an approximate correspondence. These hemodynamic indicators in the bilateral iliac grafts were quantitatively evaluated and compared in histogram form. As the cross-position ratio increased, the area-averaged weighted TAWSS of the iliac grafts showed little change (approximately 0.13 Pa), whereas the OSI decreased slightly from 0.14 to 0.13 and RRT decreased from 11.62 to 11.19.

**Fig. 10:**
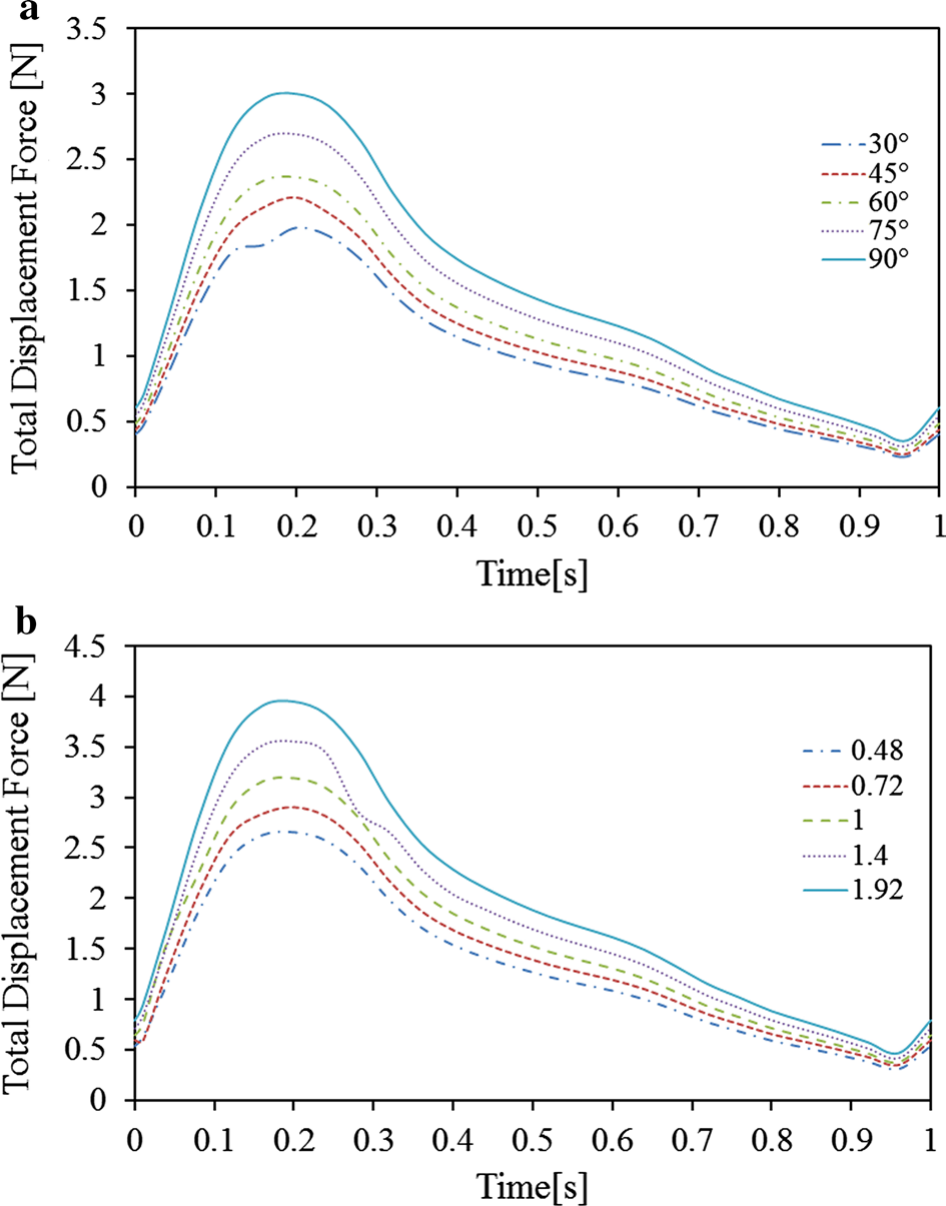
Time-variation of stent graft drag force during a cardiac cycle. **a** Effect of cross angle. **b** Effect of cross position ratio

The displacement forces acting on the stent grafts could be calculated numerically by summing forces acting normally to the stent graft walls caused by blood pressure, and viscous forces acting tangentially to the stent graft wall caused by the wall shear stress. As shown in Fig. 10, displacement force trends varied with pressure waveform trends, which have corresponded in past studies [18, 26]. The maximum displacement force occurred at the overall peak pressure of the cardiac period. The maximum displacement force gradually increased from 1.8 N to 3 N as the iliac cross angle increased and from 2.7–4 N as the cross-position ratio increased.

#### In-vitro experiment: aortic perfusion model

**Fig. 11:**
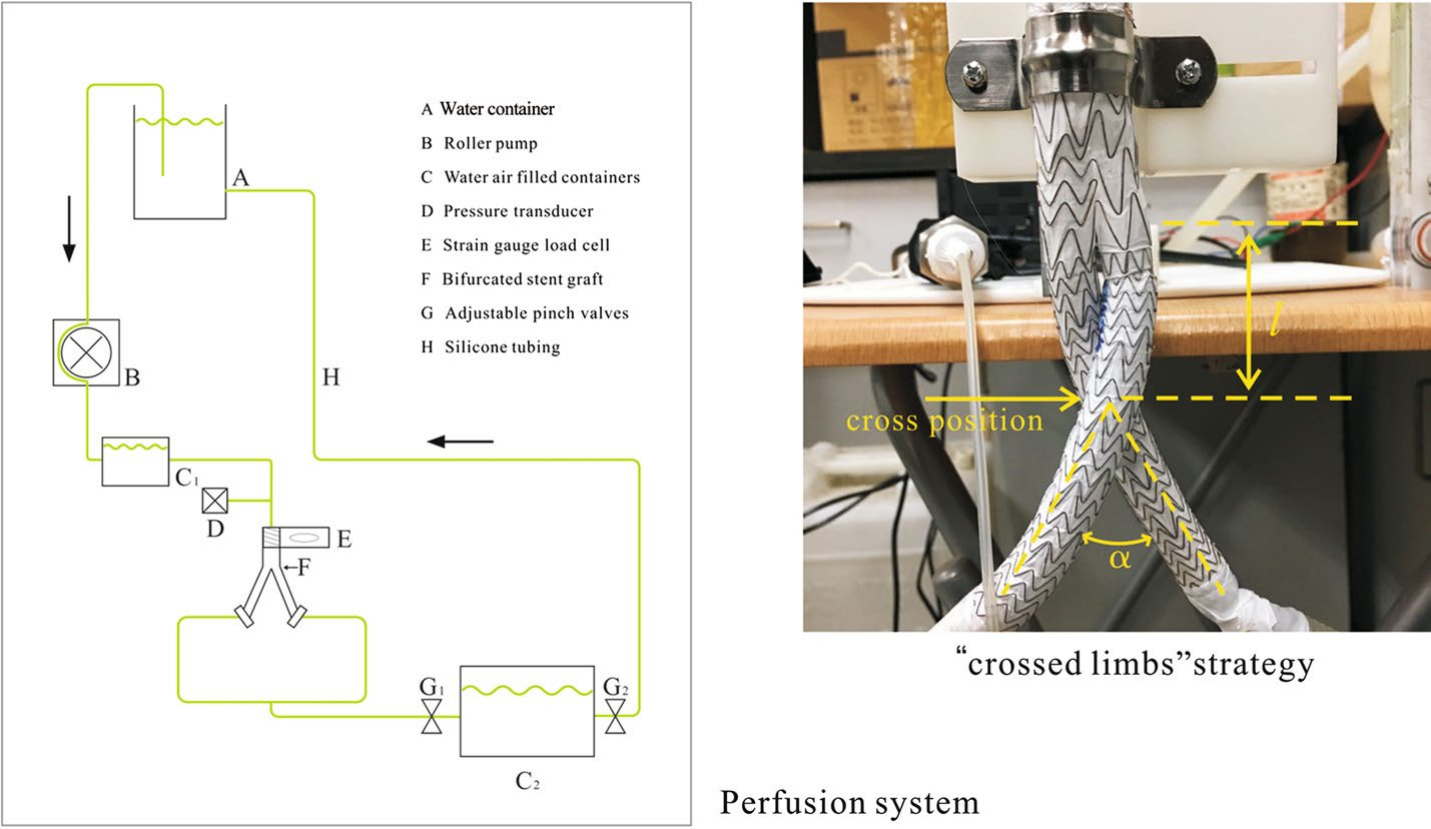
The schematic presentation of the aortic perfusion model and the BSG with indicators of cross angle (α) and cross position (*l*)

An experiment was designed in the present study to measure the displacement force acting on the BSG at different cross positions and angles. One experimental aortic perfusion system was designed to provide steady flow and simulate aortic perfusion pressure in the BSG. A mixed fluid consisting of 33.3% by volume glycerol in water (density 1.05 g/cm^3^ and dynamic viscosity 0.0033 Pa s), was used to mimic blood. Both the mixed fluid used in the experiments and the Carreau model used in the computational simulations have viscosities closely approximate blood. To simulate aortic perfusion, this fluid was perfused into a perfusion circuit consisting of a roller pump and silicone tubes at room temperature. The fluid pressure was controlled with high accuracy by adjusting the pinch valves and water levels in the containers (Fig. 11).

A BSG was inserted into the circuit with its proximal portion anchored to a strain gauge load cell via rigid connectors. The BSG used had a diameter of 26 mm at the proximal end, and 15 mm at the distal end. The BSG length was 180 mm which included the iliac graft length of 140 mm. The BSG was secured with ligatures and placed on the outside surface of the connectors to ensure that the displacement force in the vertical direction could have a maximal transfer to the load cell. The proximal and distal BSG portions were connected to the silicone tube by a soft rubber tube with highly elasticity, allowing slight displacements of the BSG, so the displacement force measurements would not be influenced. The measurement range of the load cells was 0–10 N, and calibration was performed with weights. A pressure transducer was inserted into the circuit to monitor the pressure within the iliac graft. A force monitor was connected to the load cell to display and record force values.

Displacement force measurements were conducted at perfusion pressures of 60, 80, and 100 mmHg. Perfusion pressure zero leveling and in situ calibration were performed before each measurement. Displacement force measurements were recorded every 20 s under steady flow conditions. Displacement forces are presented as mean values.

As depicted in Tables 1 and 2, the experimental results showed that the displacement force increased with increasing pressure. The displacement force gradually increased with increased cross angles. And this phenomenon tends to become more obvious when the perfusion pressure within the iliac graft reached 100 mmHg. The increase in displacement force was 0.09 N at 60 mmHg and reached approximately 0.27 N at 100 mmHg. The phenomenon was also observed as the cross-position ratio increased, and a gradually decreasing trend in displacement force was observed at all three pressure levels. The displacement force increased between 0.11 and 0.21 N with cross position variations.

**Table 1:** Flow-induced displacement forces (N) acting on the BSG under various cross angles and perfusion pressures

Cross angle	30°	45°	60°	75°	90°
60 (mmHg)	1.45	1.47	1.49	1.51	1.54
80 (mmHg)	1.62	1.63	1.65	1.66	1.7
100 (mmHg)	1.91	1.94	1.99	2.11	2.18

**Table 2:** Flow-induced displacement forces (N) acting on the BSG under various cross position ratios and perfusion pressures

Cross position ratio	0.48	0.72	1	1.4	1.92
60 (mmHg)	1.51	1.53	1.55	1.58	1.62
80 (mmHg)	1.67	1.69	1.71	1.74	1.77
100 (mmHg)	1.93	1.97	2.01	2.06	2.14

## Discussion

The crossed limbs AAA repair strategy has been often used in AAA patients with unfavorable aneurysm neck angulation or widely splayed common iliac arteries [3, 4]. Two geometric features, the cross angle *α* and cross position *l*, affect the hemodynamic performance of this strategy. This article described the construction of two crossed limbs series models with various cross angles and positions and subsequently the comparative numerical investigation of flow patterns in these models for hemodynamic performance evaluation in terms of the helical flow strength, TAWSS, OSI, RRT, and displacement force.

This study revealed that double helical blood flows, consisting of a dominant left-handed helical flow and smaller right-handed helical flow, were generated. This closely resembles results reported by Shek et al. [5, 6]. The discrepancy between the current results and theirs is that their models had a nonplanar feature whereas the current ones did not. As it is believed helical blood flows in the arterial system have physiological functions, protecting the arteries by suppressing the accumulation of atherogenic low density lipoproteins within the arterial wall [8], enhancing O_2_ supply to the artery [27], and reducing platelet/monocyte adhesion [10, 11], the crossed limbs strategy is beneficial for AAA treatment from the perspective of helical flow generation. The results obtained in this study indicate that the intensity of helical flow strength produced in the crossed limbs strategy decreased with increasing cross angles and decreasing cross position ratios. Small cross angles and low cross positions should thus be considered when implementing the crossed limbs strategy.

These results also showed that the TAWSS on the iliac artery grafts remained approximately equal as the cross angle increased, whereas the OSI and RRT decreased. These hemodynamic indicators demonstrated the same tendencies, but to negligible extents (< 3%), with cross position ratio increases. Despite these minor differences, significant high OSI and RRT strip areas appeared on the outer surface of the cross parts. High OSI strip areas were also observed in the crossed stent graft study performed by Shek et al. [5]. It has been widely recognized that high OSIs and RRTs lead to thrombosis by stimulating platelet aggregation, activating platelets, and increasing the residence time of procoagulant microparticles [28–30]. These strip areas are therefore vulnerable to thrombosis formation, potentially resulting in long-term stent graft failure.

Clinically, stent graft migration remains a well-recognized complication [26, 31]. According to the study by Li et al. [18], migration behavior could be influenced by several factors including the iliac bifurcation angle, endograft size, blood pressure, endograft wall compliance, iliac branch curvature, and neck length. For instance, in the study by Li et al. [18], the displacement force was found to increase nonlinearly with the iliac angle and blood pressure. By performing numerical simulations and conducting in vitro experiments, the results of this study not only showed that the perfusion pressure could be a significantly influential factor of migration behavior, but also that the cross positions and angles of the crossed limbs strategy are significant factors affecting BSG migration behavior. Lower cross positions and larger cross angles correlate to stronger displacement forces acting on BSGs. These results could be ascribed to the causative relationship between large cross angles or low cross positions and increases in the projected iliac artery wall surface area onto the transverse plane. Lower cross positions and larger cross angles can therefore increase the risk of stent graft migration in the crossed limbs strategy.

In the present study, although idealized crossed limbs geometric models with various cross angles and positions were constructed for simplification, it remains reasonable to draw hemodynamic parameter variation trends from the simplified results. For example, the graft wall was assumed to be rigid when it is not; however, numerical studies have shown that stent graft deformation under blood pressure is not apparent owing to its high stiffness (10 MPa) [17, 19]. Therefore, although the above simplifications might affect the accuracy of the simulation results, the major conclusions should remain the same.

## Conclusion

In summary, the strip areas of high OSI and RRT on the outer surface of the iliac artery grafts might be vulnerable to thrombosis formation. A minor cross angle and a low cross position may be optimal configurations for “crossed limbs” strategy implementation; however, low cross positions may increase the risk of migration.
